# Epigenetic Priming of AML Blasts for All-*trans* Retinoic Acid-Induced Differentiation by the HDAC Class-I Selective Inhibitor Entinostat

**DOI:** 10.1371/journal.pone.0075258

**Published:** 2013-10-08

**Authors:** Nadja Blagitko-Dorfs, Yi Jiang, Jesús Duque-Afonso, Jan Hiller, Arzu Yalcin, Gabriele Greve, Mahmoud Abdelkarim, Björn Hackanson, Michael Lübbert

**Affiliations:** 1 Department of Hematology and Oncology, University Medical Center Freiburg, Freiburg, Germany; 2 Department of Pathophysiology, Shanghai Jiao Tong University School of Medicine, Shanghai, People’s Republic of China; 3 Department of Pathology, Stanford University Medical Center, Stanford, California, United States of America; 4 University of Freiburg, Faculty of Biology, Freiburg, Germany; Georg Speyer Haus, Germany

## Abstract

All-*trans* retinoic acid (ATRA) has only limited single agent activity in AML without the PML-RARα fusion (non-M3 AML). In search of a sensitizing strategy to overcome this relative ATRA resistance, we investigated the potency of the HDAC class-I selective inhibitor entinostat in AML cell lines Kasumi-1 and HL-60 and primary AML blasts. Entinostat alone induced robust differentiation of both cell lines, which was enhanced by the combination with ATRA. This “priming” effect on ATRA-induced differentiation was at least equivalent to that achieved with the DNA hypomethylating agent decitabine, and could overall be recapitulated in primary AML blasts treated *ex vivo*. Moreover, entinostat treatment established the activating chromatin marks acH3, acH3K9, acH4 and H3K4me3 at the promoter of the *RARβ2* gene, an essential mediator of retinoic acid (RA) signaling in different solid tumor models. Similarly, *RARβ2* promoter hypermethylation (which in primary blasts from 90 AML/MDS patients was surprisingly infrequent) could be partially reversed by decitabine in the two cell lines. Re-induction of the epigenetically silenced *RARβ2* gene was achieved only when entinostat or decitabine were given prior to ATRA treatment. Thus in this model, reactivation of *RARβ2* was not necessarily required for the differentiation effect, and pharmacological *RARβ2* promoter demethylation may be a bystander phenomenon rather than an essential prerequisite for the cellular effects of decitabine when combined with ATRA. In conclusion, as a “priming” agent for non-M3 AML blasts to the differentiation-inducing effects of ATRA, entinostat is at least as active as decitabine, and both act in part independently from *RARβ2*. Further investigation of this treatment combination in non-M3 AML patients is therefore warranted, independently of *RARβ2* gene silencing by DNA methylation.

## Introduction

The treatment of older patients with acute myeloid leukemia (AML) still poses a substantial therapeutic challenge. Recently, the DNA hypomethylating agent decitabine was approved for this indication based on its significant single agent activity with a very favorable safety profile in large phase II and phase III clinical trials [1], [2]. Nonetheless, almost half of the AML patients receiving this drug do not show a response, prompting investigations of combination therapy with pan- or class-I specific HDAC inhibitors [3], or biologicals such as retinoids.

Retinoic acids (RAs) modulate complex physiological events, which trigger key steps during cellular proliferation, differentiation and apoptosis in normal and malignant cells. The beneficial effects of retinoid-based “differentiation therapy” have been clearly demonstrated in acute promyelocytic leukemia (APL): the combination of anthracycline-based chemotherapy or arsenic trioxide with all*-trans* retinoic acid (ATRA) resulted in almost complete cure rates of one of the previously most fatal subtypes of acute myeloid leukemia [4], [5]. Recently, a subgroup analysis of the AMLSG HD98D clinical trial showed that AML patients bearing NPM1-, but not FLT3-mutations had lower relapse incidence and better overall survival after induction chemotherapy combined with ATRA, suggesting a role for ATRA in the treatment of non-M3 AML [6].

The biological activity of RA is mostly mediated by all-*trans* retinoic acid receptors (RARα, β, γ) and 9-*cis* retinoic acid receptors (RXRα, β, γ), which are ligand-dependent transcription factors that bind RA response elements (RAREs) in the promoter region of target genes. In the absence of a ligand, RAR-RXR complexes repress transcription via association with a co-repressor complex, recruiting histone deacetylases (HDACs) [7], [8] and DNA methyltransferases (DNMTs) [9] and retaining a closed chromatin state. Upon RA binding, the receptors dissociate from the repressor, and transcriptional co-activators with intrinsic histone acetyltransferase (HAT) activity are recruited to the RAREs. They induce chromatin remodeling events that in turn render DNA accessible to the RNA polymerase II resulting in the transcription of RA-target genes such as *RARβ* [10].

The role of RARβ, especially of the isoform RARβ2, in mediating the growth-inhibitory effect of retinoids was demonstrated in different types of solid tumors, including breast, lung, ovarian, neuroblastoma, renal cell, pancreatic, liver, head and neck cancer [11], [12]. Exogenous RARβ in RARβ-negative cancer cells restored ATRA-induced growth inhibition and apoptosis, whereas RARβ antagonists or antisense mRNA in RARβ-positive cancer cells blocked the effect of ATRA [13]. Loss or down-regulation of *RARβ2* expression by DNA methylation [14] and histone deacetylation has been demonstrated in various cancer cell types [8], [15], [16]. The leukemic fusion proteins PML-RARα and AML1/ETO were shown to recruit HDAC and DNMT activity to the *RARβ2* promoter, resulting in gene silencing, which *in vitro* was pharmacologically reversed by HDAC and DNMT inhibitors [17], [9].

In the present study, we investigated the efficacy of entinostat, a class I specific HDAC inhibitor with antileukemic and differentiation-inducing activity in AML [18], to sensitize AML blasts to ATRA, to a degree shown with the DNA hypomethylating agent decitabine. We also asked whether this “priming” activity is linked to epigenetic reactivation of the *RARβ2* gene.

## Materials and Methods

### Cell Lines and Primary Acute Myeloid Leukemia Blasts

Kasumi-1, HL-60, NB-4, U-937, K562 and KG-1 cells were purchased from the DSMZ (GmbH, Braunschweig, Germany) and SKNO-1 [19] was kindly provided by S. D. Nimer (Sylvester Comprehensive Cancer Center, Miller School of Medicine, University of Miami, FL, USA). Cells were cultured in RPMI 1640 medium (PAA laboratories, Cölbe, Germany) supplemented with 100 U/ml penicillin/streptomycin and 10% FCS (PAA laboratories) at 37°C and 5% CO_2_. SKNO-1 cells were supplemented with 10 ng/ml GM-CSF (CellGenix, Freiburg, Germany) and 20% FCS. The cell lines were treated with different non-cytotoxic concentrations (viability >80%) of entinostat (MS-275/SNDX-275®, kindly provided by Schering AG, now Bayer Schering Pharma, Berlin, Germany) dissolved in DMSO, 5-aza-2′-deoxycytidine (decitabine, DAC, Sigma-Aldrich, Taufkirchen, Germany) dissolved in PBS, and all*-trans* retinoic acid (ATRA, Sigma-Aldrich) dissolved in DMSO. As controls, the cell lines were treated with the appropriate vehicles. Cell numbers and corresponding dead cell counts were detected with Trypan blue (Biochrom AG, Berlin, Germany).

**Figure 1:**
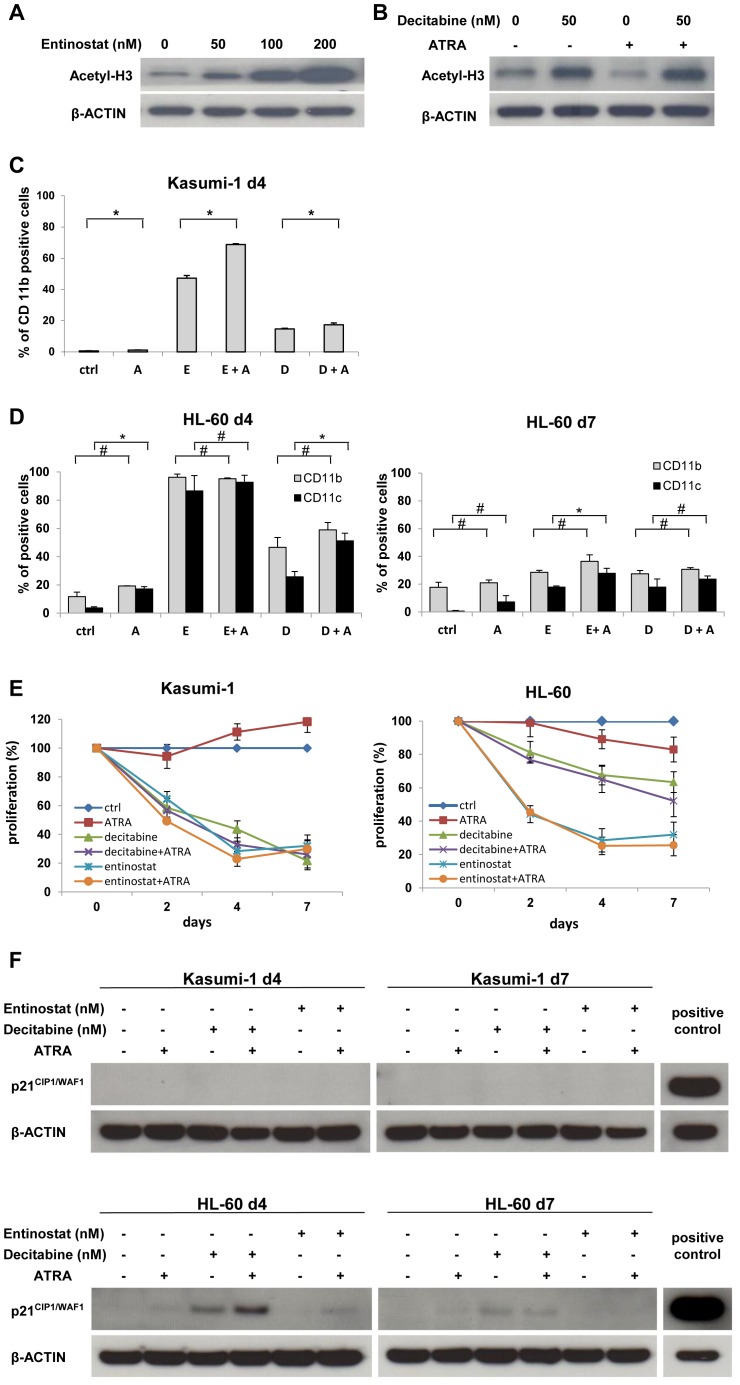
Priming effects of entinostat and decitabine for ATRA-induced differentiation in PML-RARα-negative AML cell lines. Effects of entinostat (**A**) and decitabine (**B**) on histone acetylation in Kasumi-1 cells. Cells were treated with the indicated doses of entinostat or decitabine in the presence or absence of ATRA (1 µM) as described in Materials and Methods. Whole cell lysates were subjected to immunoblot analysis for pan-histone H3 acetylation and β-actin (loading control). Effects on differentiation in Kasumi-1 (**C**) and HL-60 (**D**) cells treated with either decitabine (200 nM) or entinostat (500 nM) alone or in combination with ATRA (100 nM in Kasumi-1, 1 µM in HL-60) added on day 0. Differentiation was quantified by flow cytometry of CD11b expression in Kasumi-1 on day 4 of treatment (no quantification was feasible on day 7 due to low cell numbers) and CD11b and CD11c expression in HL-60 cells on days 4 and 7 of treatment. Bars represent the mean of 2–5 measures and error bars the standard deviation. Statistical significance by ANOVA test: * p<0.05, # not significant. (**E**) Effects of epigenetic therapy on cell growth. Kasumi-1 and HL-60 cells were treated with ATRA (100 nM in Kasumi-1, 1 µM in HL-60), decitabine (200 nM) and entinostat (500 nM) as described above. Cell proliferation was determined by Trypan blue exclusion; the percentage of cell proliferation is shown. (**F**) Induction of p21^CIP1/WAF1^ protein during prolonged cell culture. Kasumi-1 (upper panel) and HL-60 cells (lower panel) were treated with either ATRA, decitabine or entinostat for the times and with the concentrations indicated in (E). Cells were harvested after 4 and 7 days and were subjected to western blot analysis for p21^CIP1/WAF1^ as described in Materials and Methods. As loading control, β-actin was used.

Due to its short half-life (5–16 hours *in vitro* [20]) and the ability to incorporate only into replicating DNA, decitabine was added every 24 hours to the freshly replaced medium on days 0, 1 and 2 of the treatment to ensure maximum effect on DNA demethylation. Entinostat and/or retinoic acid were added to cells as a single pulse. For the differentiation and proliferation assays (Figure 1C–E), decitabine or/and ATRA as well as entinostat or/and ATRA were added as a single pulse at the beginning of treatment. Medium was replaced on days 2 and 4.

Bone marrow or peripheral blood samples of 46 newly diagnosed, untreated AML patients were kindly provided by the Tumor and Serum Repository of the Department of Hematology and Oncology, University of Freiburg. All patients signed informed consent prior to participation, and the study was approved by the ethics committee of the University Medical Center Freiburg. Patient characteristics are given in Table 1 (five AML samples treated *ex vivo*) and Table 2 (AML samples analyzed by pyrosequencing). Mononuclear cells were isolated by density gradient centrifugation. Mononuclear cells were cultured in RPMI 1640 medium supplemented with 100 U/ml penicillin/streptomycin and 20% FCS at 37°C and 5% CO_2_. Culture medium was supplemented with GM-CSF (20 ng/ml), SCF (25 ng/ml) and for cells of one of five patients also with IL-3 (6 ng/ml) and IL-6 (10 ng/ml). Primary cells were thawed, kept in culture for 48 hours and then incubated with 0.5–0.75 µM entinostat or/and 1 µM ATRA or vehicle (DMSO) for another 48 hours.

**Table 1:** AML patient characteristics.

PatientID	Gender	Age(years)	Source	WBC (Tsd/µL)	Blasts(%)	Hb(g/dL)	Plts(Tsd/µL)	FAB	Karyotype	FLT3	NPM1	fromMDS
1	F	76	PB	12.31	21	9.2	96	M1	47, XX, +13	wt	wt	no
2	m	70	PB	82.7	20	6.7	21	M4	46, XY, t(10,21)(q22;q22)	wt	wt	yes
3	m	53	PB	38.5	73	6.5	271	M7	complex, including -7	wt	n.d.	yes
4	m	46	PB	64.7	65	7.3	86	M4	46, XY	wt	n.d.	no
5	m	21	BM	52	99	9.5	76	M4	46, XY	wt	n.d.	no

PB, peripheral blood; BM, bone marrow; wt, wild type; n.d. not determined.

**Table 2:** Characteristics of AML patients analyzed by pyrosequencing.

Total	n	41	
Age at diagnosis	Median (range)	75	(66–82)
Gender	f	15	36.6%
	m	26	63.4%
BM Blasts (%)	n	31	
	Median (range)	55	(20–95)
PB Blasts (%)	n	10	
	Median (range)	20	(1–62)
WBC counts (Giga/l)	Median (range)	6.2	(0.5–241)

WBC, white blood cell counts; f, female; m, male.

### RT-PCR

Total RNA was isolated with the RNeasy Kit (Qiagen, Hilden, Germany). First-strand cDNA was generated using random hexamers and the SuperScript Reverse Transcriptase II (Invitrogen, Carlsbad, USA) following the manufacturer’s recommendations. RT-PCR was performed using HotStarTaq DNA polymerase (Qiagen) and following PCR conditions: 15 min denaturation at 95°C followed by 35 cycles of denaturation at 95°C for 30 sec, annealing at 60°C for 30 sec, extension at 72°C for 30 sec and final extension at 72°C for 5 min.

The primers *RARβ2*-RT-forward: 5′-AACGCGAGCGATCCGAGCAG-3′ and *RARβ2*-RT-reverse: 5′-ATTTGTCCTGGCAGACGAAGCA-3′ have been previously described to amplify the isoforms *RARβ2* (621 bp) and *RARβ4* (264 bp) [21]. *GAPDH* was used as reference gene. The following primers were used:

*GAPDH*-RT-forward: 5′-ACAGTCCATGCCATCACTGCC-3′;

*GAPDH*-RT-reverse: 5′-GCCTGCTTCACCACCTTCTTG- 3′.

### Flow Cytometry

Cells were washed twice with PBS and incubated with 5 µl of human mAbs (anti-CD11c-PE or anti-CD11c-APC and anti-CD11b-FITC from BD Biosciences, San Jose, CA, USA; anti-CD34-FITC and anti-CD117-PE from Miltenyi Biotec, Bergisch Gladbach, Germany) for 15 minutes, followed by PBS wash. The quantification of the cell surface markers was performed using Dako Cyan ADP flow cytometer (Beckman Coulter, Fullerton, USA) and Summit v 4.2 software (Dako, Glostrup, Denmark). Analysis and interpretation of the data were performed with the FlowJo software (Tree Star Inc., Ashland, OR, USA).

### Western Blot

Proteins were isolated with a lysis buffer containing 50 mM Tris HCl, 1% NP-40, 1% Na-deoxycholate, 150 mM NaCl, 1 mM EDTA, 1 mM PMSF, 1 mM Na_3_VO_4_ and 1 mM NaF. Protein concentration was determined by Bradford assay (BioRad, München, Germany). Equal amounts of proteins were separated using 12% Bis/Tris gels in a NuPAGE electrophoresis system (Invitrogen) and transferred to Hybond-P membranes (GE Healthcare, Uppsala, Sweden). Antibodies used for immunodetection were rabbit anti-acetyl histone H3 (Upstate, Lake Placid, NY, USA), goat anti-p21 (Santa Cruz Biotechnology, Dallas, TX, USA) and mouse anti-β-actin (Sigma-Aldrich). Proteins were detected by chemoluminescence using ECL Plus Western blotting Detection System and HyperFilm (GE Healthcare).

### Bisulfite Sequencing

Genomic DNA was isolated with the DNeasy Tissue Kit (Qiagen) and bisulfite converted using the EZ DNA Methylation Kit (Zymo Research, Irvine, CA, USA) following the manufacturer’s recommendations. The bisulfite converted DNA was amplified by PCR using HotStarTaq DNA polymerase (Qiagen) and following PCR conditions: 15 min denaturation at 95°C followed by 35 cycles of denaturation at 95°C for 30 sec, annealing at 55°C for 30 sec, extension at 72°C for 30 sec and final extension at 72°C for 5 min. The PCR products were subcloned into the pCR®2.1-TOPO® Vector (Invitrogen) and at least ten colonies were sequenced by GATC (Konstanz, Germany). The following primers were used for bisulfite converted DNA:

*RARβ2*-bis-forward: 5′-TTTAAAGTGTGGGTTGGG-3′.

*RARβ2*-bis-reverse: 5′-TCCCAAATTCTCCTTCCAAA -3′.

### Pyrosequencing

500 ng of genomic DNA was sodium bisulfite-modified using the EZ DNA Methylation Kit (Zymo Research) according to the manufacturer’s instructions. Quantitative DNA methylation was assessed by pyrosequencing as described previously [22] using the PyroMark Q96 MD system (Qiagen). Changes in DNA methylation of *RARβ2* regions 2 and 3 were analyzed after 3 and 6 days of decitabine treatment in HL-60, KG-1, U-937 and NB-4 cells, as well as in 41 AML patients. The primers used for pyrosequencing are listed in Table 3.

**Table 3:** A list of the PCR and sequencing primers used for the bisulfite pyrosequencing of the RARβ2 regions 2 and 3.

*RARβ2* region	Primer	Sequence
Region 2	Forward	5′-TGGGTTATTTGAAGGTTAGTAGTT-3′
	Reverse - Universal	5′-GTGCCAGGCTCAGGCTTCTAATCCCCCCTTTAACA-3′
	Sequencing	5′-GGGTTATTTGAAGGTTAGT-3′
	Biotin - Universal	5′-Biotin-ATCTGTGCCAGGCTCAGGC-3′
Region 3	Forward	5′- ATTTTTTGTTAAAGGGGGGATT-3′
	Reverse - Biotin	5′-Biotin-GGGATTAGAATTTTTTTATG-3′
	Sequencing	5′-GGGATTAGAATTTTTTTATG-3′

To control PCR bias, DNA methylation standards obtained by mixing unmethylated and fully methylated DNA (*in vitro* methylated with M.SssI (New England Biolabs, MA, USA)) to represent 0%, 25%, 50%, 75% and 100% DNA methylation were included in pyrosequencing analysis of each amplicon.

### Chromatin Immunoprecipitation

Chromatin immunoprecipitations were performed as described [23]. Primary antibodies used for immunoprecipitation were anti-acetyl histone H3 (AcH3), anti-acetyl histone H4 (AcH4), anti-acetyl histone H3 lysine 9 (H3K9ac) and anti-trimethyl histone H3 lysine 4 (H3K4me3), all purchased from Upstate), and normal rabbit antibody (Santa Cruz) as control antibody.

Quantitative real-time PCR was conducted on the Roche LightCycler®480 system using Light Cycler® 480 SYBR Green I Master Kit (Roche, Mannheim, Germany) to quantify DNA abundance from the immunoprecipitated *RARβ2* regions. The following primers were used to amplify region 2 (containing the RARE element) and region 3 of the *RARβ2* gene:

*RARβ2* - region 2 - forward: 5′-TGGGTCATTTGAAGGTTAGC-3′,

*RARβ2* - region 2 - reverse: 5′-CCTCCTGCCTCTGAACAG-3′,

*RARβ2* - region 3 - forward: 5′-GCTGTTTGAGGACTGGGATG-3′,

*RARβ2* - region 3 - reverse: 5′-CTTGCTCGGCCAATCCAG-3′.

All samples were measured at least in duplicates and quantified using a standard curve method. Values from each sample were normalized to 5% input and standard deviations were calculated.

### Statistics

Statistical analysis of data was performed using the SPSS v16.0 Software (SPSS inc, IBM, Chicago, USA) with ANOVA to test differences between groups and a linear regression model for drug interactions.

## Results

### Comparable Activity of Entinostat and Decitabine in Priming PML-RARA-negative AML Blasts for ATRA-induced Differentiation

In contrast to the PML-RARα -positive NB-4 cells which differentiate in response to nanomolar concentrations of ATRA [24], PML-RARα -negative, non-M3 AML blasts are much less sensitive to this physiological differentiation inducer. We treated the AML M2 myeloblast cell lines Kasumi-1 and HL-60 with ATRA and the class I-selective HDAC inhibitor entinostat.

As predicted, entinostat alone induced a dose-dependent acetylation of histone H3 (Fig. 1A), whereas ATRA treatment had no effect upon histone acetylation (Fig. 1B; notably, treatment with decitabine increased histone H3 acetylation already at 50 nM). As measured by flow cytometry on day 4 of treatment, ATRA alone induced no cellular differentiation in Kasumi-1 cells and only modest differentiation in HL-60 (<20% CD11b/c-positive cells, Fig. 1C and D, left panel). In contrast, treatment with entinostat alone robustly induced partial differentiation in Kasumi-1 and strong differentiation in HL-60 cells. The combination of ATRA and entinostat had a synergistic effect on differentiation in Kasumi-1 cells at this time point, whereas in HL-60 maximal CD11b and CD11c expression was already reached with entinostat only. In contrast, the DNMT inhibitor decitabine induced less differentiation than entinostat in both cell lines. In combination with ATRA, decitabine did not result in any priming in Kasumi-1, but had an at least additive effect on CD11c expression in HL-60 (Fig. 1D, left panel).

To find out whether the differentiating activity of entinostat and decitabine is sustained over at least two cell divisions, we determined CD11b and CD11c expression in HL-60 cells also on day 7 of treatment (Fig. 1D, right panel). Expression of both differentiation markers was significantly reduced as compared to day 4 of treatment. In Kasumi-1 cells, evaluation of differentiation on day 7 was not feasible due to low cell numbers. Treatment with both epigenetic drugs resulted in growth inhibition in both cell lines, while a “sensitizer” effect to ATRA was overall moderate (almost absent in Kasumi-1, most apparent in HL-60 only on day 7, Fig. 1E). However, we could not detect an increase in apoptosis in both cell lines, using the Trypan blue exclusion assay (see Materials and Methods, data not shown).

Since prolonged culturing may also result in differentiation-induced cell cycle arrest, we performed western blot analyses to determine whether the negative regulator of the cell cycle p21^CIP1/WAF1^ is upregulated after epigenetic treatment. As shown in Fig. 1F (upper panel), no p21^CIP1/WAF1^ protein was induced on days 4 and 7 of treatment in Kasumi-1 cells. Notably, in HL-60 cells treatment with decitabine resulted in upregulation of p21^CIP1/WAF1^ protein on day 4 (Fig. 1F, lower panel). The combination of decitabine and ATRA moderately enhanced this effect, whereas a much lower expression was induced by entinostat when combined with ATRA and none by ATRA or entinostat alone. On day 7 of treatment, upregulation of p21^CIP1/WAF1^ protein was significantly reduced.

**Figure 2:**
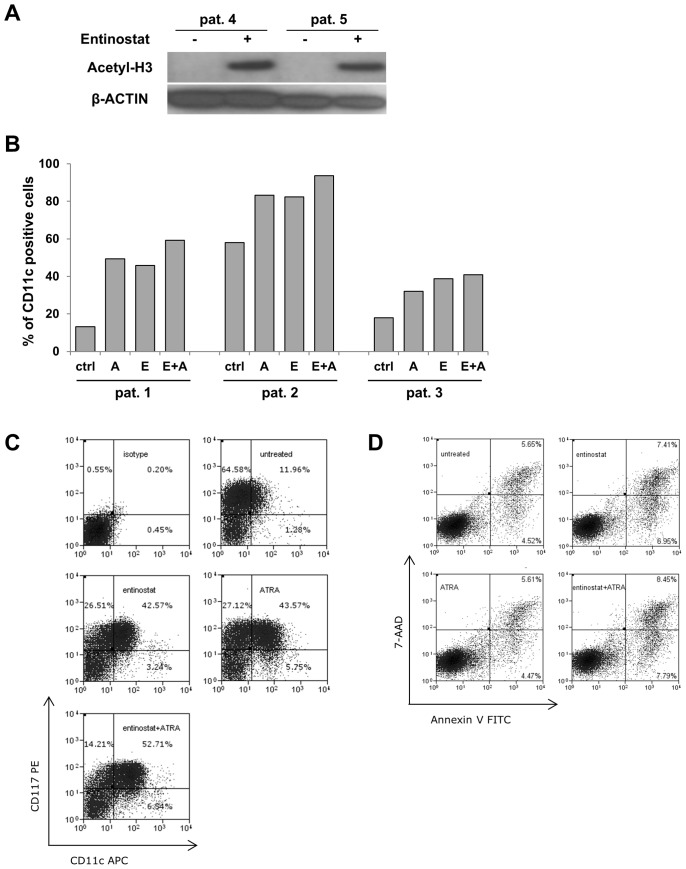
Effects of entinostat upon ATRA-induced differentiation and apoptosis in primary AML cells. (**A**) Effect of entinostat on histone acetylation in primary AML cells. Bone marrow samples from 2 patients with *de novo*, normal karyotype AML were treated *ex vivo* with a single pulse of 1 µM entinostat for 48 hours. Whole cell lysates were subjected to immunoblot analysis for pan-histone H3 acetylation and β-actin (loading control). (**B**) Effects on differentiation in primary AML blasts. Primary AML samples were treated with entinostat or/and ATRA *ex vivo* as described in Materials and Methods. Expression of the myeloid differentiation marker CD11c was quantified by flow cytometry after 48 hours of treatment. (**C**) Representative flow cytometric analysis from primary AML sample #1. Primary AML blasts were treated with 500 nM entinostat or 1 µM ATRA alone or in combination. Forty eight hours after start of the treatment, expression of the myeloid differentiation marker CD11c and the hematopoietic progenitor cell surface marker CD117 (c-Kit) were quantified using flow cytometry. PE, phycoerythrin; APC, allophycocyanin. (**D**) Effects on apoptosis in primary AML blasts. Representative flow cytometric analysis of FITC Annexin V and 7-AAD staining from primary AML sample #1. Primary AML blasts were treated as above. Cells were stained with FITC Annexin V and 7 AAD viability dye and analyzed by flow cytometry. Annexin V positive/7-AAD negative cells represent early apoptotic cells.

We next treated primary non-M3 AML blasts *ex vivo* with ATRA and/or entinostat. As expected, entinostat treatment alone resulted in strong acetylation of histone H3 in cells from 2 patients (Fig. 2A). As shown in Fig. 2B, treatment with either ATRA or entinostat markedly induced differentiation, as determined by upregulation of CD11c in 3 other AMLs (patient details in Table 1). Effects of the combined treatment were less pronounced than in the cell lines. Interestingly, an increase in CD11c expression was accompanied by reduction of CD117 expression (c-Kit) (Fig. 2C).

Enhanced growth inhibition was evident in primary AML blasts treated with entinostat and ATRA as compared to either treatment alone (data not shown). Notably, both entinostat and/or ATRA produced only minor effects on apoptosis in primary cells (Fig. 2D).

### Epigenetic Priming for ATRA-induced Differentiation is Partially Uncoupled from De-repression of a Silenced RARβ2 Gene

**Figure 3:**
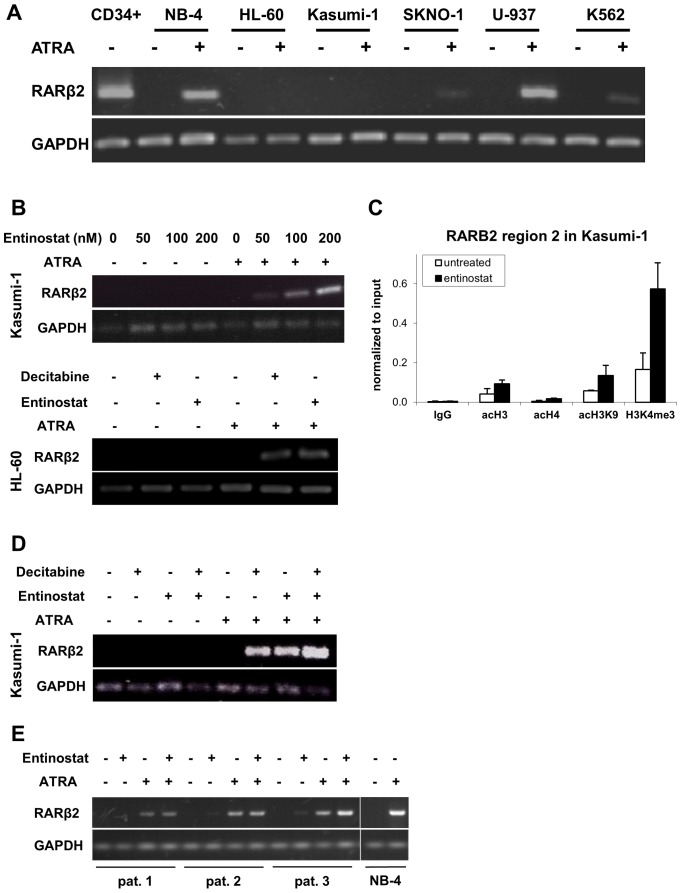
Epigenetic *RARβ2* gene silencing in myeloid leukemia cells is reversed by epigenetically active drugs. (**A**) The AML cell lines NB-4, HL-60, Kasumi-1, SKNO-1 and U-937 as well as the CML cell line K562 were treated with 1 µM ATRA for 72 hours, and expression of *RARβ2* was determined by RT-PCR. The expected band of 621 bp corresponding to the *RARβ2* isoform is shown. Normal CD34+ cells were used as a positive control for *RARβ2* mRNA expression. *GAPDH* served as a control for equal amounts of RNA. (**B**) Effect of entinostat, alone or in combination with decitabine or ATRA, on *RARβ2* expression in Kasumi-1 and HL-60 cells. *Upper panel,* Kasumi-1 cells were treated as described in Materials and Methods with entinostat and ATRA (1 µM) at the concentrations indicated. *RARβ2* and *GAPDH* (internal control) mRNA levels were assayed using RT-PCR. *Lower panel,* HL-60 cells were treated with ATRA (1 µM), decitabine (200 nM) and entinostat (500 nM) as described above. Cells were then subjected to RT-PCR as described. (**C**) Effect of entinostat on chromatin structure in the *RARβ2* gene region 2 in Kasumi-1 cells. Cells were treated with 1 µM entinostat for 24 hours, chromatin immunoprecipitation was performed for the indicated chromatin marks, followed by quantitative real-time PCR for precipitated DNA using primers specific for *RARβ2* region 2 (RARE element, see Materials and Methods). (**D**) Cooperative effects of decitabine and entinostat upon *RARβ2* re-induction. Kasumi-1 cells were treated with ATRA (1 µM), decitabine (50 nM) and entinostat (50 nM) as shown in Fig. 1A and B. Expression of *RARβ2* and *GAPDH* mRNAs was determined by RT-PCR. (**E**) Effect of entinostat or ATRA, alone or in combination, on *RARβ2* expression in primary AML blasts. Primary AML samples were treated with entinostat and ATRA *ex vivo* as described in Materials and Methods. *RARβ2* and *GAPDH* (internal control) mRNA levels were assayed using RT-PCR.

As shown in Fig. 3A, transcriptional response of the *RARβ2* gene to ATRA alone was variable between different established myeloid cell lines, with the M3 cell line NB-4 and the non-M3 cell line U-937 showing strong *RARβ2* mRNA induction in response to ATRA, whereas this induction was not detectable for Kasumi-1 and HL-60, and only weakly for SKNO-1 cells and K562 cells. However, when Kasumi-1 and HL-60 were co-treated with entinostat and ATRA, a dose-dependent *RARβ2* induction was established (Fig. 3B, upper and lower panel).

**Figure 4:**
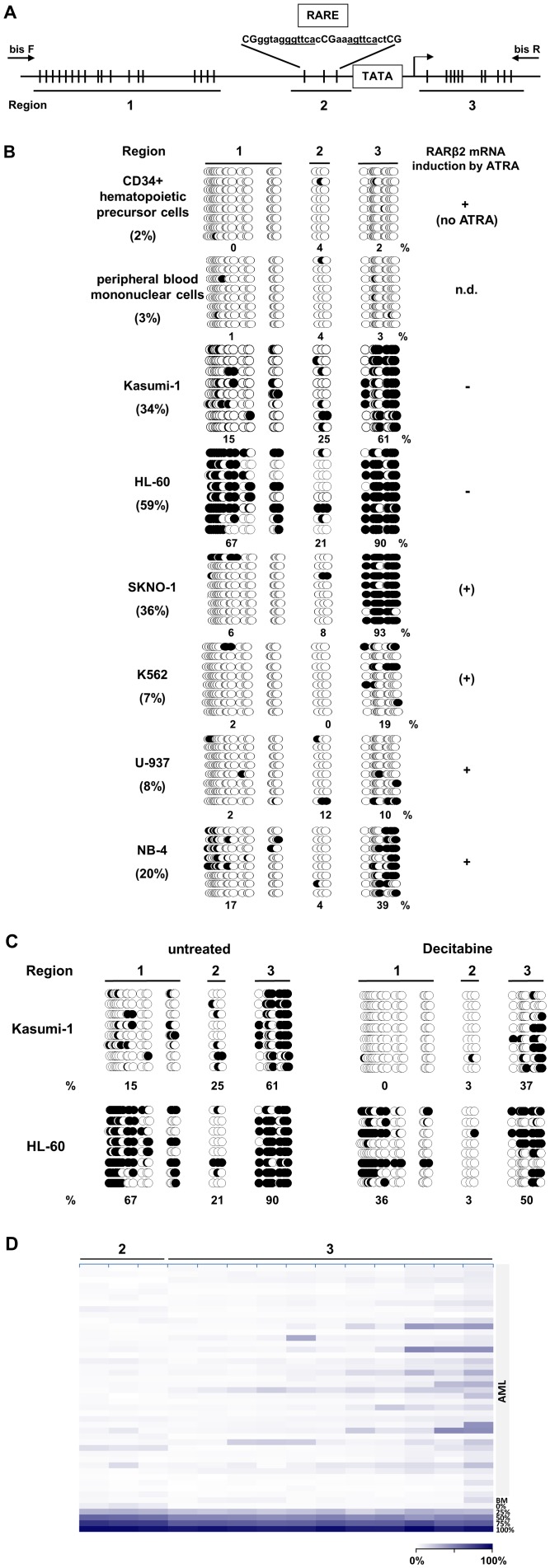
Differential methylation of the *RARβ2* promoter/exon1 in human myeloid leukemia cells. (**A**) Map of the *RARβ2* promoter region with indication of amplicons 1–3 analyzed by bisulfite sequencing (positions of forward (bis F) and reverse (bis R) primers are shown), pyrosequencing and quantitative real-time PCR of ChIP precipitates. RARE: Retinoic Acid Response Element. Vertical ticks indicate single CpGs (region 1∶18 CpGs; region 2∶3 CpGs; region 3∶11 CpGs). Arrow: transcriptional start site. (**B**) Differential DNA methylation of *RARβ2* promoter/exon1 in myeloid cells. Normal CD34+ cells, peripheral blood mononuclear cells and myeloid cell lines NB-4, HL-60, Kasumi-1, SKNO-1, U-937 and K562 were subjected to promoter methylation analyses by bisulfite sequencing of genomic DNA as described. Open circles represent unmethylated, closed circles methylated CpG sites. Percentages indicate mean methylation per region across all sequenced alleles. Region 1 was most heavily methylated in HL-60 cells, region 3 in SKNO-1, HL-60 and Kasumi-1, whereas region 2 disclosed lower overall methylation levels (25 and 21% in Kasumi-1 and HL-60, respectively, between 0 and 12% in the other cell lines). (**C**) *RARβ2* promoter/exon1 DNA methylation in human myeloid leukemia cell lines is partially reversed by DNA hypomethylating treatment. Kasumi-1 and HL-60 cells were treated with decitabine by three 24-hours incubations of decitabine (DAC) at 200 nM. Cells were harvested 48 hours after the last pulse of decitabine and subjected to bisulfite sequencing. The sequenced alleles of untreated HL-60 and Kasumi-1 cells correspond to the experiment depicted in Fig. 4B. (**D**) *RARβ2* 5′UTR/exon1 region is rarely methylated in the primary bone marrow blasts of AML patients. Quantitative DNA methylation of the *RARβ2* region 2 and 3 (as depicted in Fig. 4A) was analyzed by pyrosequencing in 41 AML patients and is shown as a heatmap. Columns represent single CpGs, each row represents a different sample. White indicates hypomethylation, dark blue indicates hypermethylation. DNA methylation standards (0%, 25%, 50%, 75% and 100% of the *in vitro* methylated genomic DNA) are depicted at the bottom of the heat map. BM, normal bone marrow.

The *RARβ2* 5′ flanking regions 2 (including the retinoic acid response element, gene map see Fig. 4A) and 3 were then analyzed for the induction of activating histone marks acH3, acH4, acH3K9 and H3K4me3 by entinostat using chromatin immunoprecipitation. All four marks were enhanced by the HDAC inhibitor in both *RARβ2* regions 2 (Fig. 3C) and 3 (data not shown). Thus entinostat may convert the *RARβ2* gene promoter to a state poised for ATRA-dependent induction. However, *RARβ2* induction was not necessary for entinostat-induced differentiation, since marked cellular differentiation was already observed upon entinostat treatment in the absence of *RARβ2* expression. Similarly, ATRA alone induced some differentiation in HL-60 cells in the absence of *RARβ2* mRNA induction.

Since decitabine also primed for ATRA-induced *RARβ2* mRNA expression in HL-60 and Kasumi-1 cells (Fig. 3B, lower panel, and D), we next performed DNA methylation analyses of the gene in both cell lines before and after DAC treatment. Compared to normal PBMC and CD34+ cells (overall *RARβ2* methylation levels 2%), the leukemia cell lines analyzed had variable degrees of DNA methylation, which was highest (59%) in HL-60, followed by Kasumi-1 and SKNO-1 (34% and 36%, respectively). Also the PML-RARα -positive cell line NB-4 had 20% methylation, whereas U-937 and K562 had only 8% and 7%, respectively (Fig. 4B). Comparable results were obtained when quantifying methylation of regions 2 and 3 using pyrosequencing (data not shown). It is noteworthy, that the degree of methylation observed in the leukemia lines often did not correlate with *RARβ2* mRNA induction by ATRA: it was hardly detectable in K562 cells but strong in U937 cells (both with similarly low methylation), conversely, it was weakly present in SKNO-1 cells, but completely absent in Kasumi-1 cells, despite quite similar hypermethylation. NB-4 cells (intermediate degree of methylation) readily showed *RARβ2* induction by ATRA.

As shown in Fig. 4C, decitabine treatment resulted in 20% reduction of overall DNA methylation in Kasumi-1 and 29% in HL-60 as compared to pre-treatment levels (confirmed by pyrosequencing, data not shown). This partial, decitabine-induced promoter demethylation was sufficient to prime both cell lines for ATRA-induced gene induction but, at least for Kasumi-1 cells, did not sensitize to ATRA-induced differentiation (see above).

Finally, we determined expression of the *RARβ2* gene in the primary blasts studied for differentiation. As shown in Fig. 3E, *RARβ2* was not expressed before *ex vivo* treatment, was weakly induced by entinostat in patients 2 and 3 and strongly induced by ATRA in all 3 patients, with some further induction with the combination treatment in patients 2 and 3. Thus the levels of transcriptional induction did not correlate with the degree of differentiation achieved. Interestingly, methylation analyses of the *RARβ2* 5′UTR and exon 1 (regions 2 and 3) by pyrosequencing of mononuclear bone marrow blasts from 41 AML patients (Fig. 4D, clinical characteristics in Table 2) and 49 higher-risk MDS patients (data not shown) did not disclose recurrent hypermethylation. Median DNA methylation frequency of 14 CpGs included in regions 2 and 3 was 2% (range 1% to 12%) in 41 AML patients, with single CpGs in region 3 showing higher DNA methylation (20% to 48%) in some AML patients. Similarly, median DNA methylation of 2% (range 1% to 7%) was detected in 49 MDS patients.

## Discussion

Considering the paramount therapeutic impact of all-*trans* retinoic acid in the cure of APL, it is difficult to conceive that this drug may be completely inactive in AMLs not expressing the PML-RARα protein. Therefore, multiple research and clinical trial groups direct their research at the elucidation of the mechanism of resistance of non-M3 AMLs to ATRA, and identification of a (genetically defined) subgroup of AML that is responsive to this drug when given in conjunction with chemotherapy [6], [17], [25]–[30] as summarized by Nowak et al. [5].

Our results support a model whereby treatment with one or two chromatin-modifying agents results in priming of cells that are relatively resistant to the cell maturation-inducing effects of the clinically very well tolerated ATRA. This differentiation therapy might be suitable to treat AML patients who are not eligible for induction chemotherapy, translating at least part of the beneficial effects of the ATRA therapy in APL to other PML-RARα-negative subtypes of AML, as proposed by Nowak et al. [5]. A multicenter randomized phase II trial by the German AML study group (AMLSG) is already underway to test the benefit of decitabine, in combination with the HDAC inhibitor valproic acid and/or ATRA in patients not eligible for induction chemotherapy (DECIDER Study, AMLSG 14-09, NCT00867672). This approach provides a therapeutic strategy whereby also other malignancies such as hormone-resistant cancers become sensitized to the physiological stimulus. This is of interest particularly in older and/or pre-treated tumor patients who would not tolerate polychemotherapy, but rather epigenetic treatment with low-toxicity drugs such as the chromatin-modifying and DNA hypomethylating agents used in this study.

Cellular differentiation was more pronounced with entinostat than with decitabine. This differentiation-inducing effect has been noted previously for myeloid leukemia cell lines [31]. Entinostat has also shown promising activity in the treatment of myelodysplastic syndromes, AML, as well as in non-small cell lung cancer [32]–[35] and other solid tumors [36]. Very recently, its HDAC inhibitor activity was also shown to be associated with a favorable clinical response in patients with breast cancer receiving entinostat and anti-hormonal therapy (ENCORE 301 phase II clinical study) [43]. The combination of entinostat with 5-azacytidine did not result in a higher response rate of MDS patients in a randomized phase II trial [3], which may be related to less DNA hypomethylation induced by the combination of both drugs or the overlapping drug schedule. Therefore, another phase II trial studying overlapping vs. sequential schedule of entinostat/5-azacytidine combination is currently ongoing (ClinicalTrials.gov, NCT01305499).

In many *in vitro* studies, pharmacologic reversal of the epigenetic silencing of the *RARβ2* gene has been described as a model to score potential therapeutic drug activity. When examining the potential of entinostat in our cell line models, we noted a quantitative “disconnect” between its ability to induce differentiation and reactivation of *RARβ2* induction by ATRA. This uncoupling was also noted when the hypermethylated *RARβ2* promoter in Kasumi-1 cells was partially demethylated by decitabine, which resulted in reactivation of *RARβ2* induction by ATRA, but not in enhanced differentiation. Tabe et al. also showed that promoter DNA methylation of *RARβ2* could be reduced by treatment with decitabine [37]. However, they were not able to show a significant induction of the *RARβ2* mRNA after treatment of Kasumi-1 cells for 24 hours with decitabine followed by ATRA. In contrast to Tabe et al., we pre-treated the cells with decitabine with 3 pulses for 72 hours and then ATRA was added, allowing a greater degree of DNA demethylation in the *RARβ2* promoter region.Several studies reported relevant *RARβ2* promoter hypermethylation in primary AML blasts as determined by methylation-specific PCR (MSP) [38], [39], [17]. However, MSP is a non-quantitative, highly sensitive assay that can lead to false positive results and therefore to an overestimation of the DNA methylation frequency as compared to a quantitative DNA methylation analysis by Mass-ARRAY or pyrosequencing [40], [41]. Using pyrosequencing, we demonstrated that *RARβ2* promoter hypermethylation is a rare event in the primary blasts of AML and MDS patients. This is in agreement with previous observations that primary malignancies exhibit lower levels of CpG island methylation as compared to matching cancer cell lines [42].

In conclusion, the class-I specific HDAC inhibitor entinostat is active not only as an antileukemic agent, but also as a sensitizer to ATRA-induced differentiation. Thus, this combination treatment warrants clinical investigations in AML and MDS patients. The entinostat - 13-*cis* retinoic acid sequence has been tested in phase I trials in patients with solid tumors with acceptable tolerance [36]. Importantly, we show that in AML, this approach should not be limited to patients with proven epigenetic silencing of the *RARβ2* gene, since differentiation induction was uncoupled from reversal of its silencing. Pharmacological reversal of DNA hypermethylation on a *RARβ2* promoter therefore does not appear to be a prerequisite for differentiation in this model.
